# Gastrostomy tube care, replacement, and removal: Expanding the registered dietitian nutritionist scope of practice to provide more comprehensive enteral nutrition support and ongoing patient‐centered care

**DOI:** 10.1002/ncp.70152

**Published:** 2026-07-14

**Authors:** Kelly McGrath, Kaitlin Schotz, Stephanie Dobak, Jane Ziegler

**Affiliations:** ^1^ Department of Clinical and Preventive Nutrition, School of Health Professions Rutgers University Newark New Jersey USA; ^2^ Department of Veterans Affairs VA Maryland Health Care System Baltimore Maryland USA; ^3^ University of Maryland Medical Center Baltimore Maryland USA; ^4^ Thomas Jefferson University Hospital Philadelphia Pennsylvania USA

**Keywords:** gastrostomy tube, home enteral nutrition, registered dietitian nutritionist, scope of practice

## Abstract

The prevalence of community‐dwelling individuals dependent on home enteral nutrition (HEN), commonly delivered via gastrostomy tube (G‐tube), continues to rise. Fragmented healthcare approaches often lead to limited follow‐up support, inadequate education, and insufficient routine G‐tube maintenance. This can lead to preventable complications, such as infection, tube dysfunction, dislodgement, and avoidable emergency department visits. The Registered Dietitian Nutritionist (RDN), already central to HEN management, is uniquely positioned to provide comprehensive G‐tube assessment and care. International models demonstrate that RDNs trained in advanced practice roles to deliver G‐tube care, including balloon G‐tube (BGT) replacement, can improve access, enhance patient satisfaction, and reduce healthcare utilization and costs. Expanding the RDN scope of practice to include G‐tube management represents an opportunity to improve continuity of care for HEN consumers while addressing gaps in healthcare access and reducing health care system burden. This narrative review describes current practices for routine BGT care and exchanges and explores the benefits of RDN‐led G‐tube management. Three U.S.‐based institutional approaches to credentialing RDNs in G‐tube care and replacement are described. In addition, key implementation components, such as stakeholder engagement, policy development, competency‐based training, and interdisciplinary collaboration, are highlighted. Standardized training pathways, broader support, and further research on outcomes are needed to support wider implementation of this advanced practice role.

## INTRODUCTION

Enteral nutrition (EN) support refers to the administration of nutrition, hydration, and/or medications through a tube into a functional part of the digestive tract.^1^ EN is a critical intervention to prevent or treat malnutrition and optimize nutritional status for patients with a functional gut who are unable to meet nutritional needs orally.^1^ In the inpatient setting, a growing number of registered dietitian nutritionists (RDNs) are acquiring specialized EN management privileges, including nasogastric and nasoenteric tube placement.^2^ While nasogastric and nasoenteric tubes are generally used for short‐term EN support, gastrostomy tubes (G‐tubes) are indicated for long‐term EN support, typically exceeding 4 to 6 weeks.^3^, ^4^ In 2013, an estimated 437,882 U.S. pediatric and adult patients received long‐term EN in the home setting.^5^ Despite this already large number, home enteral nutrition (HEN) need is projected to grow alongside EN technology advancements and an aging population.^5^

HEN consumers require ongoing monitoring to prevent metabolic complications, such as malnutrition, dehydration, and electrolyte abnormalities.^6^ However, mechanical complications (such as peristomal skin issues) and tube‐related complications (such as clogging, breakages, or inadvertent dislodgement) also occur, leading to avoidable emergency department (ED) visits.^7^ Although quality of life often improves in the weeks following inpatient discharge, patients starting HEN and their caregivers frequently report considerable stress and emotional burden related to ongoing care demands.^8^ These individuals express a need for structured follow‐up and clear guidance on whom to contact when issues arise.^8^ Clinical practice guidelines recommend at least quarterly follow‐up to assess HEN efficacy, monitor HEN tolerance, and reinforce education and support.^9^ Sadly, HEN consumers and their caregivers have reported limited confidence in their primary care provider's ability to manage HEN, such as ordering supplies and formula, or addressing tube complications.^10^, ^11^

G‐tube management may be delegated to Registered Nurses (RNs) in outpatient and home settings.^12^ Like other clinicians, RNs require training and demonstrated competency to perform this skill.^12^ However, the pervasive shortage of RNs in the U.S. has led to increased workload and reductions in experienced, competent nursing staff.^13^ In these conditions, omissions in care can occur.^13^ In the case of G‐tube management, the authors noted a lack of routine care for HEN consumers in their respective institutions, partly due to RN staffing shortages or lack of training in this skillset. Similarly, physicians and advanced practice providers (APPs) often face high patient volumes and have limited capacity to deliver lower‐reimbursed services, such as routine HEN management, G‐tube exchanges, and peristomal skin assessments.^11^, ^14^ Additionally, patients with mobility limitations may require specialized transport to medical appointments, creating an additional financial and logistical burden for both the patient and the healthcare system. There is also evidence suggesting that HEN consumers prefer a centralized, single point of care to manage their nutrition and device‐related needs.^8^, ^11^

RDNs play a central role in HEN management by monitoring nutritional status, evaluating tolerance and adequacy of feeding regimens, ordering HEN formula and supplies, and performing components of the nutrition‐focused physical examination, which may include assessment of enteral access devices (EADs) and surrounding skin integrity.^1^, ^7^ Given their expertise and frequent patient contact, some RDNs may be well‐positioned to adopt advanced practice roles in outpatient and home settings, including routine G‐tube exchanges and site care. In this capacity, RDNs can serve as a consistent, accessible point of contact and deliver comprehensive, patient‐centered HEN care. However, implementation of this advanced scope of practice (SOP) should be driven by institutional need, patient volume, available resources, and the presence of qualified RDNs with appropriate training and demonstrated competency. Not all practice settings will have a need for RDN‐led G‐tube management. Furthermore, the number of credentialed RDNs should be sufficient to meet patient needs, while remaining limited enough to allow regular performance of these skills to maintain procedural competency.

This review summarizes current evidence and practices related to G‐tube care and replacement, examines the clinical and economic benefits of RDN‐led gastrostomy management, and discusses considerations for expanding the RDN SOP in this area. In addition, we describe three U.S.‐based models for credentialing RDNs in G‐tube care and replacement, highlighting key elements of program development, including stakeholder engagement, policy creation, competency‐based training, and interdisciplinary collaboration.

## GASTROSTOMY TUBE DESIGN AND COMPLICATIONS

G‐tubes vary by method of placement and internal retention mechanism. Endoscopically placed G‐tubes have a solid mushroom‐shaped internal fixator, which is removed using traction.^15^ In contrast, laparoscopically or radiographically placed G‐tubes contain a sterile water‐filled balloon internal fixator, which is deflated prior to removal. Once the gastrostomy tract has matured, generally 4–6 weeks following initial G‐tube placement, BGTs are the replacement tube of choice when G‐tube exchange is required.^16^ BGTs can be safely removed and replaced at bedside without the need for endoscopic or surgical intervention and carry a very low risk for bleeding or disruption of the established tract.^17^, ^18^, ^19^, ^20^ Several bedside techniques may be used to confirm proper intragastric tube placement. These include aspiration of gastric contents or water irrigation to evaluate for resistance or patient discomfort.^16^ Additionally, a colored fluid infused into the existing G‐tube and aspirated back through the newly placed BGT can confirm correct placement through confirmation of the color change of gastric content.^20^, ^21^ If these methods are not successful, a contrast study is necessary to confirm placement.^16^

G‐tube and BGT‐ related mechanical complication rates have been reported to be as high as 30%.^22^ The most common mechanical complications are tube occlusion, breakage, and dislodgement, as well as skin concerns, such as the hypergranulation of stomal tissue or localized infection.^6^, ^23^, ^24^ Clinical practice guidelines recommend G‐tubes with solid internal fixators be exchanged approximately 1 year after placement, whereas BGTs require replacement every 3 to 6 months per manufacturer specifications.^7^, ^18^, ^19^ Failure to replace BGTs within this interval increases the risk of balloon degradation and tube dislodgement, which may result in interruptions to nutrition, hydration, and medication delivery.^22^ Fortunately, most of these complications can be effectively managed in the home or outpatient setting with appropriate monitoring, intervention, and patient education.^6^, ^25^ Timely interventions in these settings, such as routine BGT exchanges and concurrent peristomal skin assessment, can prevent unnecessary ED visits and reduce healthcare utilization.^6^, ^25^

## THE NEED FOR GASTROSTOMY TUBE EDUCATION

Patients with newly placed G‐tubes should receive comprehensive education prior to discharge to the community.^26^, ^27^ Education sessions often cover the administration of formula, water flushes, and medications through the tube, as well as operation of the enteral infusion pump when applicable.^9^, ^27^, ^28^ Initial education should also address essential aspects of G‐tube management, including stoma and tube care, recognition and initial management of common complications, processes for obtaining supplies, and expectations for follow‐up care.^27^ Best practice guidelines recommend incorporating a return demonstration or other competency‐based assessment to confirm patients or caregivers can safely manage EN in the post‐acute setting.^6^, ^9^, ^29^ Coupled with training, clear guidance on whom to contact for questions or urgent concerns is a critical component of discharge preparation.^30^

Despite these recommendations, the hospital discharge process is frequently fast‐paced and overwhelming, limiting patients’ ability to comprehend and retain complex care instructions.^31^ Inpatient education sessions are often fragmented, time‐limited, and insufficient in scope, leaving gaps in patient and caregiver preparedness for managing HEN.^32^, ^33^ Indeed, patients with newly placed tubes may be unaware of potential complications until they occur in the home, and only then realize they do not know how to manage them.

While a growing body of literature supports the use of online resources such as YouTube for HEN information and education, there are important limitations with these tools.^34^ Not all patients possess the digital proficiency, access to technology, or health literacy required to effectively utilize and contextualize web‐based information. Inadvertently, patients may deviate to non‐evidence‐based content that has not been developed or vetted by qualified healthcare professionals, potentially leading to inappropriate or unsafe practices.^34^ Ultimately, HEN management requires individualized assessment and guidance that cannot be fully replicated through standardized online resources. Many HEN consumers benefit from ongoing support provided by familiar clinicians, such as an RDN with HEN experience, with whom they have an established relationship.^11^

## THE ROLE OF THE RDN IN GASTROSTOMY TUBE REPLACEMENT AND MANAGEMENT

Although the American Society for Parenteral and Enteral Nutrition (ASPEN) has not issued a position statement specifically addressing RDN‐performed BGT replacement, ASPEN and the Academy of Nutrition and Dietetics (AND) have recognized that nutrition support practice evolves through additional education, training, and demonstrated competency. While AND has published standards describing competent, proficient, and expert levels of practice for RDNs in nutrition support,^35^ ASPEN has recently developed educational resources (YouTube™ videos) and clinical guidance related to BGT replacement for both clinicians and caregivers.^36^, ^37^

Given the relatively low risk associated with BGT exchange in a mature tract, multiple studies have demonstrated that appropriately trained healthcare professionals, as well as HEN consumers and caregivers in the home setting, can safely perform routine BGT replacements.^7^, ^19^, ^20^ However, consumers and caregivers may be unable or unwilling to perform these procedures independently. As such, there remains a critical need for accessible, trained clinicians to provide routine BGT care and replacement.

Routine follow‐up with an RDN allows for tailored adjustments to the HEN regimen and can extend to longitudinal monitoring of G‐tube function, site integrity, and timely tube exchanges. To date, several international models highlight the effectiveness of this approach. For example, in the United Kingdom (U.K.) and Australia, various healthcare systems have established nutrition support teams (NSTs) in which the RDN plays a central role in G‐tube education, replacement, care, and maintenance.^25^, ^38^, ^39^, ^40^ These programs provide consumers with a centralized, accessible resource for HEN management and more consistent follow‐up. Reported benefits include improved consumer satisfaction and quality of life, enhanced nutritional outcomes, reduced healthcare costs, and fewer ED visits.^20^, ^38^, ^40^ Furthermore, enabling consumers to receive ongoing care in the home setting may reduce the psychological burden associated with G‐tube placement and long‐term HEN management.^25^

Australian and U.K.‐based RDN‐led NSTs have also demonstrated considerable cost savings, for not only the consumer, but also the healthcare system. These savings are driven by reduced EN supply waste, fewer ED visits, decreased hospital readmissions, and reduced need for specialty services, such as radiology and endoscopy.^25^, ^38^, ^39^, ^40^ In one prospective study, a dietitian‐led HEN team reduced hospital readmissions related to G‐tube complications from 23% to 2%.^25^ Another large prospective study evaluated the outcomes of ten advanced practice RDNs leading G‐tube care across five Australian institutions. Over the 2‐year observation period, no adverse events were reported, and cost savings across sites exceeded $185,000 AUD.^39^ Although the U.S. payer system differs substantially from those of Australia and the United Kingdom, avoidable G‐tube‐related ED visits, hospitalizations, and specialist referrals similarly contribute to healthcare expenditures in the U.S., suggesting that comparable cost savings may be achievable through implementation of RDN‐led G‐tube management programs.

In a qualitative study, Stanley and Borthwick conducted semi‐structured interviews with six community‐based RDNs who extended SOP to include G‐tube care and replacement in the home setting.^40^ These RDNs noted that role expansion was largely driven by unmet needs for G‐tube care in the community, particularly within healthcare systems characterized by limited staffing, funding, structure, and coordination of care.^40^ Participants also described how clinical role substitution facilitated more efficient use of healthcare resources.^40^ In the context of nursing shortages and elevated cost of physician‐delivered procedures, training RDNs to perform routine BGT exchanges provided a practical and sustainable alternative, reducing reliance on specialist services.^40^ Taken together, these findings further support that advanced practice RDN‐led G‐tube management is a safe, patient‐centered approach that can improve access to care while reducing healthcare costs.

## RDN SCOPE OF PRACTICE

**Figure 1:**
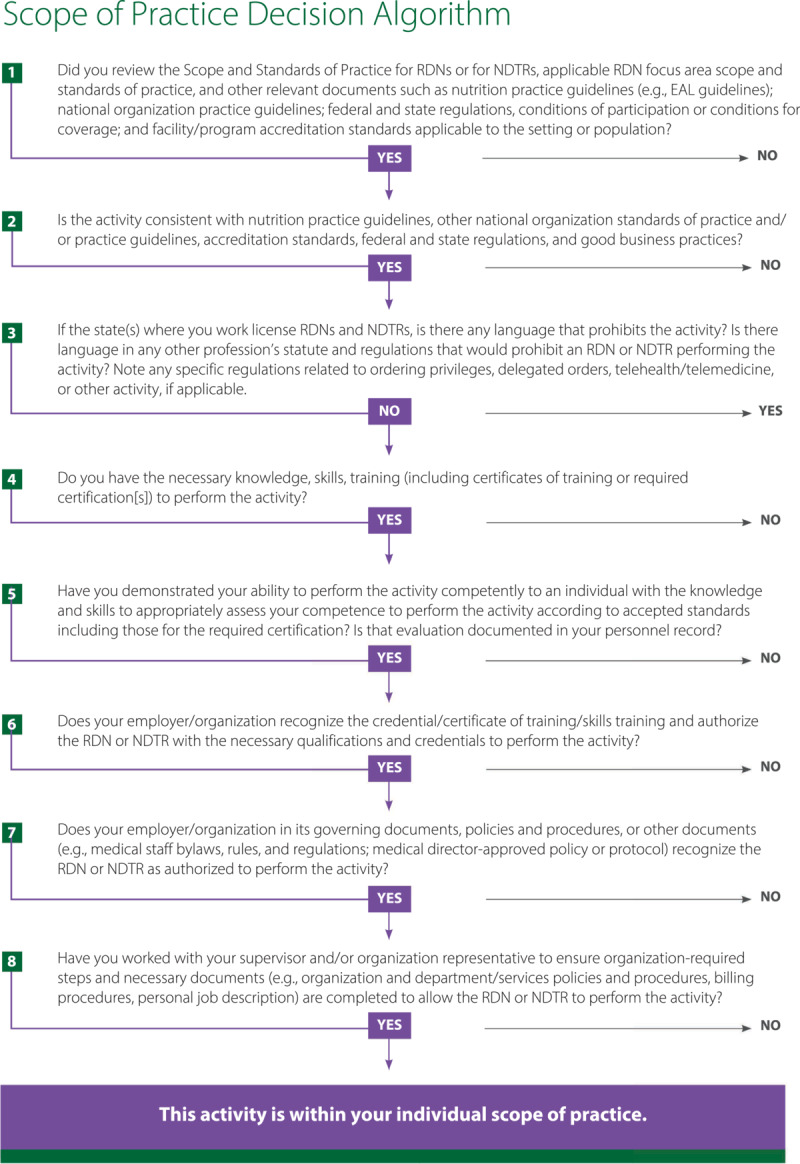
Scope of practice decision algorithm.^42^ EAL, Evidence Analysis Library; NDTR; Nutrition and Dietetics Technician, Registered; RDN, Registered Dietitian Nutritionist. Permission to reproduce has been obtained.

The Commission on Dietetic Registration's (CDR) *Revised 2024 Scope and Standards of Professional Practice (SOPP) for RDNs* recognizes that RDNs may expand their individual SOP through additional education, training, demonstrated competency, and institutional support.^41^ An individualized RDN SOP must also align with facility policies as well as applicable state laws and regulations.^41^ To guide this process, the CDR developed the Scope of Practice Decision Algorithm (Figure 1), which assists RDNs in evaluating whether a specific skill or procedure is appropriate within their individual SOP and competency level.^42^ This algorithm identifies key steps and entities which should be considered to ensure that the proposed expanded RDN SOP is supported by both the profession and the institution.^42^

Importantly, the RDN SOP is not static, but individualized based on professional preparation, competency, and institutional privilege. The CDR's *Revised 2024 SOPP* specifically acknowledges advanced practice responsibilities related to EADs, noting that management of complex conditions may require “specific knowledge, skills, and specialized training,” including procedures such as “insertion of nasoenteric feeding tubes.”^42^ The document further states that, with appropriate privileges or delegated authority, RDNs may provide “nutrition‐related services,” including “inserting and monitoring nasoenteric feeding tubes” and “performing and educating on home enteral nutrition or infusion management services.”^42^

As noted above, one established example of expanded RDN practice is the placement and management of nasoenteric feeding tubes. Although historically considered outside the RDN SOP, this role has been increasingly adopted across healthcare institutions and has been associated with improved clinical outcomes, including reduced delays in nutrition initiation, shorter hospital length of stay, and lower rates of malnutrition‐related complications.^43^, ^44^ Similar to the process undertaken for RDN‐led nasoenteric tube placement, advanced practice roles involving BGT management would also benefit from structured education, competency‐based training, and institutional and regulatory approval to ensure safe and effective care delivery.^2^ For institutions seeking to implement RDN‐led G‐tube management, clear policies, procedures, and competency‐based training frameworks are essential to ensure safe and effective practice. This article describes three U.S.‐based institutional approaches to credentialing RDNs in G‐tube care and BGT exchanges, highlighting key considerations for successful program implementation.

## INSTITUTIONAL POLICY AND RDN TRAINING DEVELOPMENT

**Figure 2:**
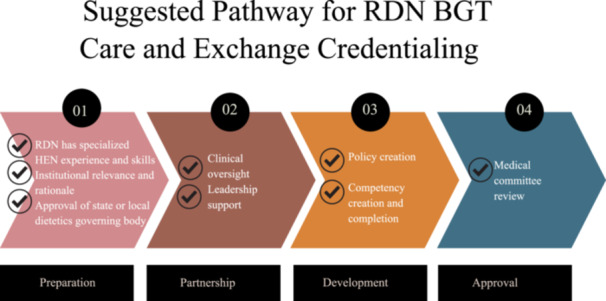
Suggested pathway for RDN BGT care and exchange credentialing. Legend: BGT, balloon‐retained gastrostomy tube; HEN, home enteral nutrition; RDN, registered dietitian nutritionist.

Development of an RDN‐led BGT management and exchange program requires a structured, interdisciplinary approach which includes preparation, partnership, policy development, competency‐based training, and institutional approval (Figure 2). Key considerations for implementation are outlined below.

### Preparation

Institutions should evaluate the clinical need and relevance of an RDN‐led BGT service, including identification of the target patient population(s) most likely to benefit. Literature reviews should be used to support the development of best practices for the desired procedures, workflows, and patient outcomes. Collaborating with team members who currently provide G‐tube care to the patient population of concern may help to generate support for the proposed service. Collecting baseline institutional data, such as G‐tube related ED utilization, tube‐related complications, delays in care, or specialist referral burden, may help establish rationale for program development and support future outcomes assessment.

In parallel, the institution should refer to established methods used for other disciplines to confirm that RDNs possess the foundational HEN knowledge and clinical experience necessary to pursue advanced practice privileges in BGT management and exchange. Additionally, organizations and/or the RDN should engage the appropriate state licensure or regulatory board, when applicable, to confirm alignment between the proposed responsibilities and current state practice regulations.^42^

Table 1 outlines the resources utilized to demonstrate the RDN SOP, develop a training and competency plan, and ultimately, establish institutional policies in three institutions.^17^, ^41^, ^42^, ^45^, ^46^, ^47^ This is not an exhaustive list, however, and may be used in part or in its entirety, based on the appropriateness for institutional applications.

**Table 1:** Resources for RDN scope of practice and G‐tube management.

**Scope of practice**
CDR 2024 Scope and Standards of Professional Practice for the RDN (CDR)^41^
AND‐CDR Scope of Practice Decision Algorithm (CDR)^42^
State Board of Dietetic Licensure (varies by state)
Liability Insurance resources (varies by state)
**Competency protocol**
Literature review for BGT Clinical Practice Guidelines and exchange procedures
StatPearls Gastrostomy Tube Replacement^20^
Clinical Key long shaft G‐tube exchange training protocol (Elsevier)^45^
Clinical Key low profile G‐tube exchange training protocol (Elsevier)^46^
ASPEN Bedside Feeding Tube Placement Competency Tool Checklist^47^

Abbreviations: AND, Academy of Nutrition and Dietetics; ASPEN, American Society for Parenteral and Enteral Nutrition; BGT, Balloon‐retained Gastrostomy tube; CDR, Commission on Dietetic Registration; RDN, Registered Dietitian Nutritionist.

### Partnership

Successful implementation requires strong interdisciplinary collaboration and clinical oversight. Support from RDN leadership as well as physicians, APPs and RNs involved in the care of HEN patients is essential to ensure safe integration of the service into existing care models. Early stakeholder engagement may also facilitate institutional acceptance and streamline the approval process. Partnerships may also help to identify subject matter experts (SMEs) who can provide the supervised training required for the RDN to establish competency in BGT exchanges.

Since regulations governing advanced procedural responsibilities vary by state and institution, consultation with legal counsel, risk management, compliance, and professional licensing boards may be necessary during program development. Institutions should also review professional liability and malpractice coverage for both the individual RDN and the organization. Expanded procedural responsibilities may require modification of existing coverage, addition of specific privileges within credentialing documents, or confirmation from malpractice carriers that G‐tube management and exchange activities fall within the covered RDN SOP. Early involvement of risk management and insurance representatives can identify potential liability concerns and help establish appropriate safeguards, training requirements, and documentation standards. Additionally, individual professional liability insurance may provide an extra layer of protection.

Reimbursement considerations are similarly important for long‐term program sustainability. Depending on the practice setting and payer mix, institutions may identify billing pathways for clinical services, procedural care, and associated supplies. Collaboration with billing, coding, and compliance teams can help determine available reimbursement mechanisms and ensure documentation supports billing requirements. By engaging legal, regulatory, risk management, and reimbursement stakeholders early in the planning process, institutions can identify barriers, establish appropriate oversight, and create a sustainable framework for advanced RDN practice in G‐tube management.

### Development

Institutions should develop a comprehensive, institution‐specific policy outlining indications, contraindications, escalation procedures, documentation standards, and clinical oversight requirements for BGT care and replacement, including those led by RDNs. A formalized training plan and competency evaluation process should accompany the policy. Existing educational resources and competency checklists, such as those available through ASPEN, Elsevier Clinical Skills, or StatPearls, may serve as useful frameworks for adaptation.^17^, ^45^, ^48^

Competency‐based training should include both didactic instruction and supervised procedural experience.^49^ Given the procedural nature of BGT exchange, education and assessment are ideally provided by clinicians with expertise in G‐tube placement and replacement, such as gastroenterologists, surgeons, interventional radiologists, and nurses with advanced training.^39^, ^50^

### Approval

Following development of the policy and competency framework, institutional approval should be obtained through the appropriate clinical and administrative review processes. This often includes presentation to multiple committees for feedback and approval. Supporting materials may include baseline institutional data, evidence from the literature, anticipated clinical and operational benefits, and details regarding training and competency validation.

Once the policy and competency process is approved and incorporated into facility policy, the RDN can complete the training process and implement BGT management in the desired patient population.

After implementation, institutions should consider collecting post‐intervention workflow and outcome metrics to compare with baseline data. Monitoring outcomes, such as complication rates, ED utilization, patient satisfaction, adverse events, and healthcare costs, may help justify continued program support and further advance the role of the RDN in specialized EAD management.

## INSTITUTIONAL IMPLEMENTATION EXAMPLES

The following section describes the approaches used to establish RDN‐led BGT management and replacement privileges across three U.S. medical institutions. Given the variations in setting and patient population, we describe each setting below, as well as the impetus to the credentialing request. Table 2 describes the departments which supported the process and the SMEs who provided the procedural training. The resulting institutional policies are then described, which include requirements for RDN experience, competency assessment, and necessary state and institutional approval.

**Table 2:** G‐tube competency and credentialing processes at three healthcare sites.

Aspects of policy development	Site A (Home Care)	Site B (Outpatient Oncology/GI Clinic)	Site C (Outpatient ALS Clinic)
Supporting departments	Clinical Nutrition Department	Clinical Nutrition Department Gastroenterology Department	Neurology Department
	Gastroenterology Department		Surgery Department Clinical Nutrition Department
	Medical Director		
Clinical training designee	Gastroenterology providers	Gastroenterology providers	General Surgery providers
Required RDN experience and advanced practice credentials	≥5 years HEN support experience	≥5 years HEN experience	No specific requirements
	CNSC certification	CNSC or CSO certification	
	RDN‐AP certification or advanced degree (MS, DCN, or equivalent)		
	BLS certification		
State and institutional approval	State licensure board	State licensure board	State licensure board
	Legal and regulatory/compliance including liability insurance review	Legal and regulatory/compliance including liability insurance review	Legal and regulatory/compliance including liability insurance review
		Ambulatory Leadership Committee	Nursing Committee
		Medical Executive Committee	Policy and Accreditation Committee
		Reimbursement team	Medical Executive Committee
Training protocol and competency development	Observe 3–5 BGT exchanges by SME	Observe 3–5 BGT exchanges by SME	Observe ≥3 BGT exchanges by SME
	Successfully exchange 3 BGTs under SME observation	Successfully exchange 5 BGTs under SME observation	Successfully exchange ≥3 BGTs under SME observation
	Maintain competency by exchanging 4 BGTs annually	Maintain competency by exchanging 1 BGT annually under SME observation	Maintain competency by exchanging ≥3 BGTs annually
	Maintain BLS certification		

Abbreviations: ALS, amyotrophic lateral sclerosis; AND, Academy of Nutrition and Dietetics; BGT, balloon gastrostomy tube; BLS, Basic Life Support; CDR, Commission on Dietetic Registration; CNSC, Certified Nutrition Support Clinician; CSO, Certified Specialist in Oncology; DCN, Doctor of Clinical Nutrition; ED, emergency department; EN, enteral nutrition; G‐tube, gastrostomy tube; GI, gastrointestinal; MS, Master of Science; RDN, registered dietitian nutritionist; RDN‐AP, registered dietitian nutritionist‐advanced practice; SME, subject matter expert.

Site A is a community‐based program within a large healthcare system which serves patients who are homebound and require supportive care. Interdisciplinary teams serve these patients by performing home visits at predetermined discipline‐specific intervals and are provided vehicles for travel. Home safety inspections occur upon admission to this program and are performed routinely by the team. HEN consumers enrolled in this program comprise a small proportion of the overall population served, less than 10% since 2020. However, these consumers often required ongoing HEN education and specialized transportation to scheduled G‐tube exchanges at the affiliated gastroenterology clinic or other community hospitals. Specialty transportation incurs expenses for the HEN consumer, the affiliated clinic, and the healthcare system at large. Gastroenterology department appointments historically were scheduled by the HEN consumer based on their preference and proximity to the affiliated clinic. Those who experienced G‐tube failure frequently required ED visits to community hospitals, often resulting in transfers to larger hospitals which were better equipped to manage G‐tube concerns.

In this program, the RDN follows HEN‐dependent patients quarterly to monitor tolerance, weights, and biochemical parameters, and provides ongoing education regarding HEN administration techniques and G‐tube site care among established patients. Given the vulnerability of these HEN consumers and the demonstrated barriers in obtaining necessary management of G‐tube complications, the RDN began tracking G‐tube related ED visit frequency, incidence of clogging and other tube malfunctions, as well as stomal skin concerns. The RDN then investigated the process for obtaining competency to perform BGT management and exchanges in the home. This data was used to justify an RDN‐led BGT management team, which includes the program Medical Director and patient providers. Once a standardized policy and competency process were developed and approved, and RDN competency was demonstrated, RDN‐led routine BGT exchanges were implemented for approximately 10 patients. HEN consumers presenting to the program with solid‐bolster G‐tubes were scheduled with the gastroenterology team for replacement with BGTs for subsequent management in the home by the RDN.^28^ It is noteworthy that clinical practice guidelines for the frequencies of BGT exchanges and HEN surveillance visits are similar (roughly every 3 months), such that additional visits beyond those dictated by the program are not required for routine BGT management.^9^, ^19^ The RDN is available for troubleshooting BGT concerns outside of this schedule; however, this has not been necessary. The RDN continues to track BGT care in this program, noting stomal skin concerns, tube malfunction, G‐tube related ED visits, and successful BGT exchanges performed in the home. Thus far, 28 BGT exchanges have been performed in the home by the RDN with no recorded adverse events, complications, or ED visits.

Of note, additional consideration should be taken for community‐based G‐tube services to address the safety and logistical burden placed on the RDN. Home visits require assessment of neighborhood and environmental safety, adherence to institutional policies regarding community‐based care, and access to communication and support resources when concerns arise. Travel time and transportation costs must also be considered, as these may reduce clinical productivity compared with clinic‐based encounters. Institutions implementing home‐based RDN‐led G‐tube services should evaluate staffing models, geographic service areas, scheduling efficiency, and reimbursement structures to ensure that the benefits of increased patient access outweigh the associated personnel and transportation costs.

Site B is a large urban academic medical center serving a substantial outpatient gastroenterology and oncology population. Prior to implementation of an RDN‐led gastrostomy management program, several gaps in care were identified by the RDN, including inconsistent HEN education at hospital discharge, absence of a proactive tube replacement schedule, and lack of a centralized point of contact for HEN‐related concerns. Frequent ED visits for G‐tube complications, particularly tube dislodgements and peristomal concerns, were also observed. Further assessment revealed that RNs, APPs, and oncology providers caring for these patients often lacked specialized training in G‐tube maintenance and replacement. Although gastroenterology providers possessed the necessary expertise, their clinical time was largely devoted to advanced endoscopic procedures, limiting their capacity to provide routine G‐tube care and proactive tube exchanges. Interventional Radiology providers were rarely able to accommodate outpatient bookings, such as G‐tube concerns, on short notice.

To address these challenges, the RDN partnered with the Clinical Nutrition Director and gastroenterology providers to develop an institutional policy and competency‐based training supporting RDN‐led G‐tube management and routine BGT replacement. Given the size and complexity of the patient population, a provider‐delegated order set was developed in collaboration with electronic medical record specialists, allowing referring providers to delegate specified G‐tube management activities to the advanced practice RDN. Approval from the state licensing board, as well as the institution's legal and regulatory/compliance teams, ambulatory nursing leadership, and medical executive leadership, was obtained. It was determined that the proposed G‐tube management functions were part of the RDN SOP.

Additional infrastructure was established in partnership with nursing leadership, including a dedicated clinic space and standardized supply procurement process to facilitate inventory management and budgeting. To support program sustainability, a billing workflow was developed in collaboration with the reimbursement and compliance team. This included utilization of non‐physician provider billing codes for clinical services, as well as product‐specific codes for G‐tube supplies, enabling revenue generation while expanding access to specialized G‐tube care. The single advanced practice RDN has provided care for more than 150 HEN consumers in 14 months of operation. To date, there have been no avoidable ED visits or complications recorded. At times, non‐routine observations, such as concern for buried bumper syndrome, infection, or non‐healing stomal sites, have required escalation to the provider or wound care team.

Site C includes a multidisciplinary amyotrophic lateral sclerosis (ALS) clinic representing 12 different clinical professions to collaboratively address the various needs of people living with amyotrophic lateral sclerosis (pALS). When dysphagia or other factors inhibit pALS’ ability to consume adequate oral nutrition, discussions of G‐tube placement for EN are recommended.^51^ As ALS progresses, mobility becomes impaired, and transportation becomes problematic, it is particularly important to avoid preventable ED visits for G‐tube related issues. Often in ALS clinics, the RDN manages the EN care plan and assesses the G‐tube and peristomal skin.

At Site C, the neurologist and nurse practitioner lacked specialized training in G‐tube exchange and management. Although the surgery team who placed the G‐tubes was willing to exchange G‐tubes, the surgery clinic operated concurrently with the ALS clinic. In an effort to consolidate care within the existing ALS care team, the RDN was deemed the clinician best positioned to triage G‐tube related issues and lead routine BGT exchanges. In 2025, the ALS RDN, with the support of the ALS and surgery teams, sought institutional privileges to perform BGT exchanges and manage minor peristomal skin issues (e.g., hypergranulation tissue, excoriation). Initial removal of solid bolster G‐tubes continue to be performed by surgery providers, and these G‐tubes are being replaced with BGTs such that subsequent exchanges can be done by the RDN.^52^ An institutional policy and competency checklist was created and approved by three different institutional committees. At the time of this writing, the RDN has independently and successfully exchanged eight G‐tubes.

Regarding costs and supplies, the ALS RDN obtains replacement G‐tubes through the pALS's durable medical equipment (DME) company. The process for procedural revenue is currently being investigated. While the RDN triages G‐tube and peristomal skin concerns, any higher‐level concerns are escalated to the surgery team. Data collection on the impact of this role substitution in G‐tube management is currently underway.

## CONCLUSION AND FUTURE DIRECTIONS

Considerable international literature supports the successful implementation of advanced RDN practices in G‐tube care.^38^, ^39^, ^40^ RDN‐led G‐tube services have demonstrated meaningful clinical and economic benefits, including reductions in ED utilization, hospital readmissions, specialist referrals, and EN supply waste.^25^, ^38^, ^39^ Patient‐centered G‐tube management provided by an RDN may improve continuity of care, accessibility, convenience, and overall satisfaction while maintaining patient safety. Expanded procedural responsibilities may also enhance professional satisfaction for RDNs and further strengthen recognition of the dietetic profession as an integral component of interdisciplinary nutrition support efforts.

In 2022, the Accreditation Council for Education in Nutrition and Dietetics (ACEND) revised its standards to require dietetic interns to “explain the steps involved and observe the placement of nasogastric or nasoenteric feeding tubes; if available, assist in the process of placing nasogastric or nasoenteric feeding tubes.”^53^ At present, however, ACEND standards do not include exposure to G‐tube replacement or management. Incorporating G‐tube care and replacement into future educational standards or advanced training opportunities could help expand the number of U.S. RDNs prepared to perform BGT exchanges. Development of standardized training pathways and competency‐based credentialing processes would further support practice specialization and facilitate broader implementation of RDN‐led G‐tube services.

Expansion of advanced procedural competencies may also benefit the profession by increasing the technical and clinical distinctiveness of RDN practice. As healthcare delivery evolves and artificial intelligence increasingly influences aspects of nutrition care, acquisition of specialized procedural skills may enhance the versatility and value of the RDN within interdisciplinary care models.^54^, ^55^ Advanced competencies such as BGT management may also represent a meaningful avenue for professional growth and career development.

As with any clinical procedure, BGT exchange carries inherent risks. It is important to acknowledge individual competency and escalation pathways. At this time, BGT exchanges are not regularly performed by RDNs and are not included in dietetic curriculums. Therefore, institutions implementing RDN privileges for BGT exchanges and G‐tube management should establish protocols outlining circumstances warranting referrals to more experienced providers, such as physicians, APPs, or nurses (including wound and ostomy nurses).

As noted, the three institutions mentioned in this article have differing competency requirements, reflecting differences in patient populations and clinical workflows. Competency programs should balance patient safety and clinician risk with implementation feasibility. Overly burdensome training requirements may limit implementation, particularly given that non‐medical caregivers are routinely taught to exchange G‐tubes in the home setting.^56^, ^57^

This manuscript primarily discusses routine BGT replacement in established tracts. While some non‐routine situations (such as intractable tube clogs, valve incompetence, or balloon bolster malfunction) may be appropriate for management by a trained RDN, more complex complications (such as suspected buried bumper syndrome, peristomal infection, or significant peristomal leakage or bleeding) should prompt escalation to a qualified provider. In addition, certain circumstances, such as site infection, may require a prescription from a provider.

While trained RDNs may triage patients with G‐tubes and help address gaps in access to routine G‐tube management, expanded RDN SOP should not be viewed as a replacement for interdisciplinary care. Ultimately, the safest and most effective approach to BGT management is a collaborative model involving RDNs, physicians, APPs, RNs, and wound care specialists. This shared interdisciplinary strategy allows for a comprehensive approach to education, discharge planning, routine and non‐routine BGT management, and appropriate staff utilization.

Persistent workforce shortages, rising healthcare costs, and increasing demand for HEN services highlight the need for innovative care delivery models. Role substitution through utilization of appropriately trained RDNs in G‐tube management represents one potential strategy to improve efficiency, expand access, and reduce healthcare burden. Future research should evaluate the prevalence of RDN‐led BGT replacement in the U.S., associated safety and clinical outcomes, patient and clinician‐reported experiences, economic impact, and barriers to implementation of this advanced practice role. In addition, studies should evaluate operational factors, such as RDN travel requirements, workforce capacity, staff safety, and the cost‐effectiveness of home‐based versus clinic‐based gastrostomy management models.

## AUTHOR CONTRIBUTIONS

Kelly McGrath, Stephanie Dobak, and Jane Ziegler contributed to conception and design of the manuscript. Kelly McGrath, Kaitlin Schotz, and Stephanie Dobak contributed to literature review and analysis, and drafted sections of the manuscript. All authors read, critically revised, and approved the final manuscript and agree to be fully accountable for ensuring the integrity and accuracy of the work.

## CONFLICT OF INTEREST STATEMENT

None declared.
